# Behavioral responses of wild chimpanzees toward a juvenile that suddenly lost its animacy due to a fall accident

**DOI:** 10.1038/s41598-023-43229-0

**Published:** 2023-10-04

**Authors:** Masaki Shimada, Wataru Yano

**Affiliations:** 1 Department of Animal Sciences, Teikyo University of Science, 2525 Yatsusawa, Uenohara, Yamanashi 409-0193 Japan; 2 Department of Biology, National Defense Medical College, 3-2 Namiki, Tokorozawa, Saitama 359-8513 Japan

**Keywords:** Animal behaviour, Anthropology

## Abstract

Detailed observations of animal reactions to a collapsed individual in wild are rare but essential to debates about the perception of death by nonhuman animals, including chimpanzees. A male juvenile chimpanzee named Volta, a member of the M group in the Mahale Mountains National Park, fell from a tall tree and was temporarily incapacitated, suffering a severe concussion and nasal bone fracture. However, Volta showed signs of gradual recovery. We compared the behavior of other chimpanzees towards Volta with the previous reports on the behavior towards collapsed or recently dead group members. We found that behaviors towards Volta were similar to those observed towards collapsed or dead members. These included other-regarding behaviors and aggressive behaviors, and notably, licking of Volta’s blood, which has not been previously reported. Adult males tended to be in close proximity to Volta for longer periods than adult females. The social situation with adult males including alpha male, surrounding Volta likely influenced the behavior of other individuals. Exploring the state of recovery of the injured individual, by closely approaching, directing various behaviors, and observing the reactions of the victim, and demonstrate tolerance and consideration towards the victim.

## Introduction

For the wild chimpanzees (*Pan troglodytes*), crashers from high altitudes undergo high-energy impacts that can result in death or serious injury^1–5^. For example, a healthy adult male in Gombe, Tanzania, fell from a tree, fractured his neck, and died immediately^1^. Anatomical findings of bones collected in the field have shown ante-mortem fractures due to falls and healing marks^3,6,7^. However, the frequency of crashes in wild chimpanzees from high altitudes is considered extremely low^7,8^. For example, in the Tai National Park of Côte d'Ivoire, 12 cases of falls were observed during a 17-year long-term study, and only one death was confirmed to have occurred from a fall^2^.

The response of chimpanzees, one of the most phylogenetically close animals to humans, to the loss of animacy in other individuals for whatever reason has been of interest because it may provide clues elucidating how they recognize the loss of others^9,10^. The signs of lethal conditions in wild individuals include bleeding and loss of consciousness. When encountered with a bleeding, but conscious individual, it has been reported that chimpanzees may lick the blood of injured individuals^10–12^. However, few cases of loss of consciousness have been observed among other chimpanzees.

De Marco and colleagues reviewed 13 studies that reported on the behavior of captive and wild chimpanzees toward collapsed and inanimate members, that is, alive but unconscious and unable to move voluntarily^9,13^, who were not immediately identifiable as dead, except for the records of mothers’ attitudes towards their dead offspring^10^. Behaviors, such as stay nearby/visitation, rough treatment, peer, display, touch/manipulate, and sniff, are mentioned in more than half of the studies and considered to be commonly observed; groom, warning call, and distress call are in less than half (more than four) studies and considered to be occasionally observed; and other behaviors, such as attack and swat flies, are in one or two studies and considered to be rarely observed. There have been no previous reports of chimpanzees licking collapsed or inanimate group members. Although chimpanzees commonly exhibit strong emotional reactions, exploratory behaviors, and other-regarding behaviors towards collapsed and inanimate members, the behavioral patterns are not uniform and vary among groups and among individuals^10,14^.

Unlike other diseases and accidents, falls can cause sudden fatalities without the signs of illness or injury. How would chimpanzees react to an individual who suddenly loses animacy due to a head injury caused by a fall? Whether in humans or animals, it is only in essentially one-time-only events such as falls that empathy and sympathy for the victim are likely to become apparent^15,16^. However, in the wild, it is usually impossible not only for human observers, but also for conspecifics to determine whether an animal is dead or inanimate, will soon die, or may recover in near-future, only through temporary observation from outside^10,17^. Therefore, other individuals need to detect the animacy of the victim for a certain period of time^18^. Clarification about changes in the reactions of surrounding individuals toward an individual who was healthy and suddenly becomes incapacitated after an accident and changes in the victim’s condition over time is crucial to understand animacy detection, empathy, sympathy, and the perception of death in chimpanzees. However, researchers rarely encounter accidental falls, and only a few cases have been reported^1^. Especially, information is limited on how chimpanzees in the wild react to other individuals in a collapsed state^10^. Reproduction of such accidents is impossible in a laboratory, which is why detailed reports of such cases from the wild and captivity are valuable.

This report describes and characterizes the reactions of chimpanzees to a juvenile male member who fell from a high altitude and temporarily lost the animacy and ability to behave normally. By comparing their behavior to that reported in previous studies, we aimed to add insight into their perceptions of victims and their cognitions related to death.

## Results

### Observations

At 9:40 a.m. on September 2, 2022, we and an assistant began following Nkombo. Nkombo and Volta were traveling as members of a relatively large party that included the alpha male of the M group, Teddy.

**Figure 1:**
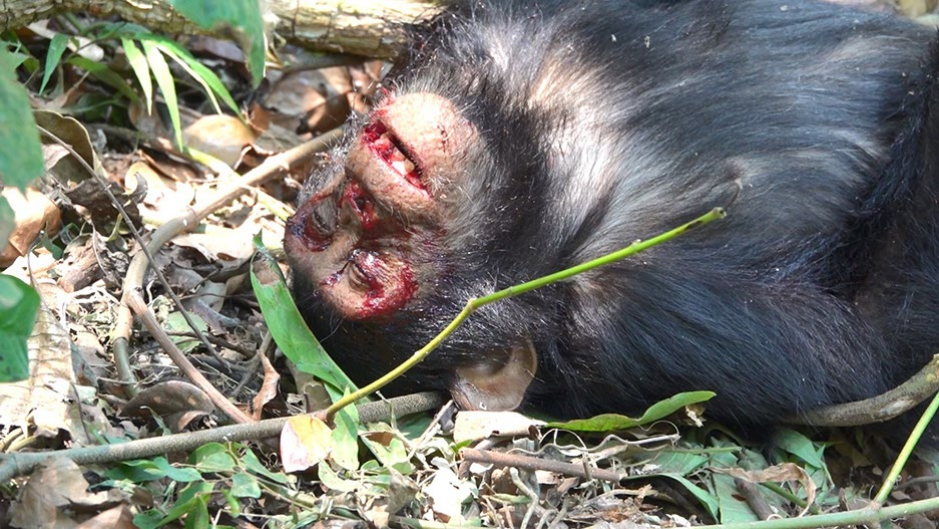
Volta at 12:41 p.m., just after confirmation of the accidental fall; blood on the face has not dried.

At 12:24 p.m., Nkombo and Volta were resting on the ground near a tall tree of *Pseudospondias microcarpa* near the tourist camp. At 12:37 p.m., when an adult male, Ichiro initiated a charging display on the ground and rushed toward Nkombo and Volta, they immediately ran up to the tall tree of *P. microcarpa*. The assistant observed that Volta fell from the tip of a tree branch, approximately 7 m above the ground. However, the moment at which Volta’s crashed or landed could not be confirmed. At 12:41 p.m., we found Volta lying on the ground on his back with both the eyes closed and the mouth half open, gulping and having hiccup-like convulsions every few seconds. Although the blood was noted on the orbits of the eyes, upper eyebrow ridges, cheeks, and gums, which appeared to have been drained from both nasal passages, no trauma to the head or other body parts was observed (Fig. 1). Both hands were holding the vines on the ground.

**Figure 2:**
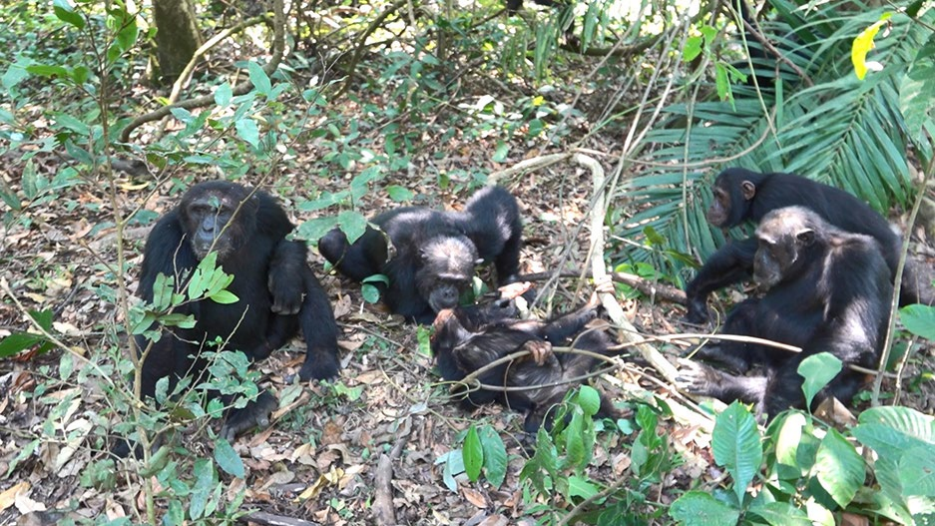
At 12:44 p.m., Orion licking Volta’s face while holding a dead leaf with blood on it. Figaro, Omaly, and Nkombo were sitting on the tree about 5 m away from Volta and watching him. The crash site is on the right of the photo, approximately 2 m away from Volta.

The discovery point of Volta was approximately 2 m away from the crash site in a straight line, with several fresh blood-stained fallen leaves and other debris scattered between them. When we began observing Volta, an adult male, Orion stood quadrupedally approximately 3 m from Volta, holding a piece of dead leaf with fresh blood on it; Ichiro was sitting facing Volta, and another adult male, Carter, was also standing quadrupedally watching Volta. At 12:42 p.m., Orion approached Volta’s left hand, neck, and face, and sniffed and licked blood from Volta’s face. Subsequently, Ichiro and Carter smelled Volta’s left hand, stomach, neck, etc. Carter picked up a dead leaf of *Saba comorensis* with blood found near the crash site, smelled it, and immediately abandoned it. Orion sat in close proximity to Volta, holding the blood-covered dead leaf. At 12:44 p.m., when a part of Volta’s face made slight contact with Ichiro’s face due to convulsions, Ichiro moved away slightly, Carter and Orion peered into and smelled Volta’s face, and Orion slightly licked the blood on it (Fig. 2). Several chimpanzees were distributed in the trees and on the ground around the 10-m perimeter of Volta, but did not vocalize at all. Young males Figaro and Omaly, and Nkombo were sitting on vines and branches approximately 5 m from Volta, looking down on him.

At 12:45 p.m., Carter swatted away a fly flying near Volta. Volta removed his left hand from the vine. Carter stood quadrupedally, looked at the Volta’s face, smelled it, and licked him. Volta remained on his back, raised his head slightly off the ground, and grabbed Carter’s hair around the neck with his left hand.

At 12:46 p.m., Carter walked away and a juvenile male Oz approached in his place, peered into Volta, sniffed him, and Ichiro, who was sitting in close proximity to Volta, reached out with his left hand and poked Oz’s head; Oz screamed and ran away. Ichiro and Orion walked away from Volta’s side. Volta remained lying on his back in the same place, when an adult female, Christina, approached from the north, stood quadrupedally, peered into Volta’s face, and sat in close proximity to him. The alpha male of the M group, Teddy, and an adult male, Emory, approached Volta simultaneously, standing quadrupedally and peering into Volta’s face and stomach. Teddy piloerected. Orion licked Volta’s face.

At 12:47 p.m., Ichiro licked Volta’s stomach and face. Four adult males (Teddy, Emory, Orion, and Ichiro) and one adult female (Christina) sat close to Volta. When Teddy peered into Volta’s face, Volta blinked his eyes, whimpered, showed his grimace, and reached out with his left hand to touch Teddy. Orion and Teddy peered into Volta’s face simultaneously and Orion licked Volta’s face. Emory sniffed Volta’s hair. An adult male, Darwin, approached from the south with piloerection, and chimpanzees crowded around Volta. Orion and Emory broke off just before Darwin approached, and Christina emitted pant-grunts and walked away.

At 12:48 p.m., Darwin and Teddy simultaneously initiated charging display. Ichiro silently left the site. Teddy shook a vine and charged it bipedally. Darwin stood bipedally around Volta and shook the vine with Teddy. Volta sat up on the ground on his own as Teddy finished his display. Volta walked slightly but stumbled and fell on his back. Teddy touched Volta’s back and peered into his hips. Simultaneously, Ichiro and Orion approached and licked Volta’s face. Volta sat down and reached out with his left hand to touch Teddy’s mouth. Teddy bit the finger of Volta’s left hand. The sitting position caused blood to flow out of both the nostrils of Volta.

**Figure 3:**
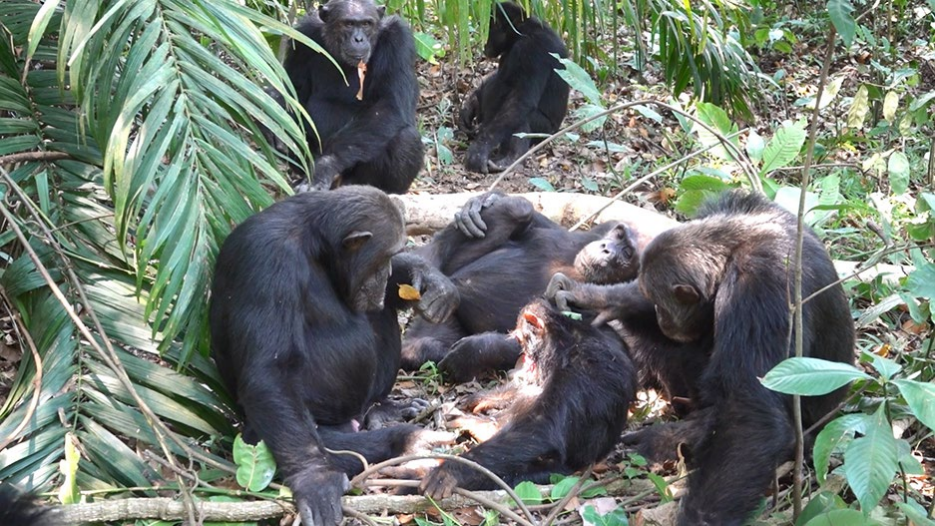
Teddy (right) grooming Volta in a sitting position. Christmas (left) looking at blood on a dead leaf. Orion (upper left) sitting 3 m away from Volta and holding a dead leaf with blood on it in his mouth.

At 12:50 p.m., an adult male and the son of Christina, Christmas, approached and lifted Volta’s chin slightly to inspect the face and oral cavity. Volta had been gulping. At 12:51 p.m., Teddy groomed the back of Volta. AZ and Nkombo were sitting in a tree 3 m above the ground, and Omaly and Emory were sitting 5 m above the ground, looking down at Volta and others. At 12:54 p.m., Teddy groomed the head of Volta, and Christmas picked up a dead leaf with blood on it and stared at it for a moment (Fig. 3). At 12:57 p.m., an estrous adult female, Effie, sniffed and left Volta.

At 12:59 p.m., Teddy stopped grooming Volta and laid down. Darwin and Emory were seated 3 m away from Volta. At 13:00, Christmas groomed Teddy. At 13:02, Nkombo moved to the ground, approached Volta, peered into Volta, and explored the blood on the ground. Volta was lying on his back with the mouth half open. At 13:03, Nkombo sat 5 m away from Volta. Christmas touched Volta’s left hand. Volta laid on his back with his left foot touching the forehead of Christina.

**Figure 4:**
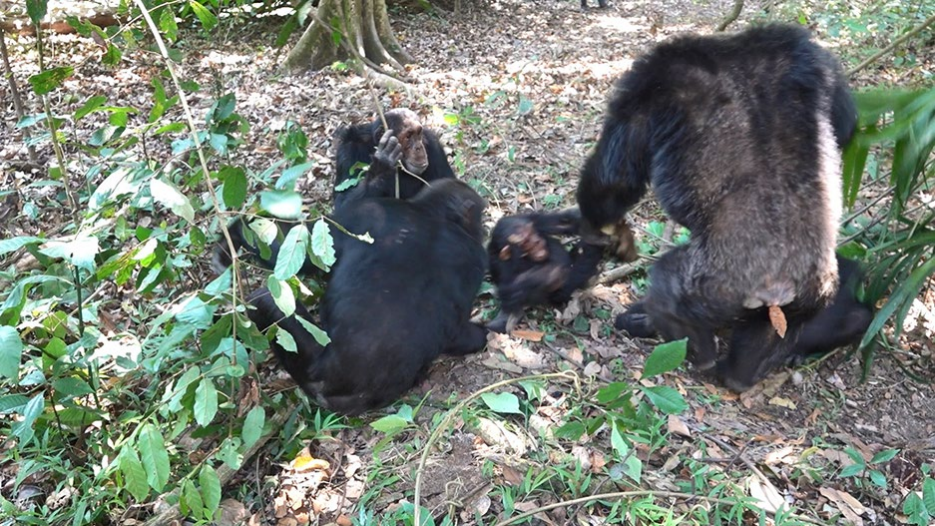
Teddy (right), suddenly bristling with hair, grabbed Volta’s right leg and began a charging display.

At 13:07, Teddy, who was sitting in close proximity to Volta, suddenly started charging display silently with his fur bristling, grabbed Volta’s right leg and dragged Volta slightly (Fig. 4), shook a branch of the palm tree, and let go of Volta. The surrounding individuals escaped and Volta walked on his own, grimacing and wobbling for approximately 2 m. The surroundings became calm within a few seconds. Volta sat down, and Orion approached and licked Volta’s face. At 13:10, when Volta opened his mouth wide, Orion placed his right index finger in Volta’s mouth. Volta was gagging terribly and looked sick.

At 13:17, Teddy, who was lying approximately 2 m away from Volta, suddenly started charging display, grabbed Volta’s left arm, and dragged him for 1 m. Teddy sat on the ground approximately 3 m away from Volta. Approximately 5 m away from Volta, Darwin started charging display, slapping the back of Nkombo, and Nkombo screamed. Volta approached Teddy, wobbled, emitted a pant-grunt, and sat in contact with Teddy. At 13:18, Orion began grooming Volta and continued until 13:37. At 13:38, Volta bled and vomited blood from the nose and mouth. Orion groomed Volta from 13:42 to 13:47 and from 13:59 to 14:05. Teddy groomed Volta from 13:44 to 13:49. At 14:05, as Christmas approached Volta, Teddy charged on Christmas. Volta emitted a pant-grunt toward Teddy, who sat nearby, and approached and extended his hand to Teddy. Teddy gently bit the index finger of Volta.

At 14:10, Volta vomited blood out of his mouth and Orion watched him. At 14:11, Orion and AZ started charging display, and Teddy also started display and charged toward Orion and Volta. Teddy stood bipedally and shook the bushes around Volta. Volta emitted a pant-grunt toward Teddy, but Teddy ran away without stopping. After charging display by the alpha male and others, many members of the party simultaneously walked off toward northeast. Volta had a lot of saliva dripping from his mouth, was wobbling, walked quadrupedally, moved 5 m, and sat down. At 14:12, Orion looked at the face and mouth of Volta. Orion sat 5 m away from Volta. The voices of the group shifted away from the northeast. Subsequently, Volta repeatedly approached Orion on foot. At 14:43, Orion, who was sitting 5 m away from Volta, repeatedly shook a vine. When Volta staggered closer to Orion, Orion groomed Volta. Thereafter, Orion repeatedly moved and sat about 5–10 m away from Volta, and when Volta approached, Orion repeatedly groomed Volta for a short time.

**Figure 5.**
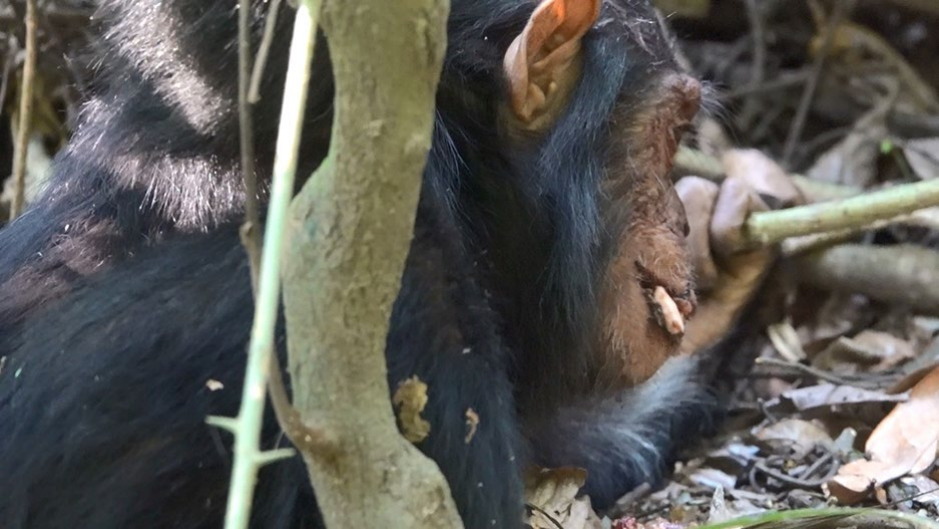
Volta vomiting out stomach contents from his mouth.

At 15:34, Orion, who was sitting 10 m away from Volta, walked away toward northeast, and no other chimpanzees were observed around Volta. At 16:02, 16:14, and 17:28, Volta vomited his stomach contents and became limp, leaned against a tree, laid down, and occasionally gasped (Fig. 5). At 17:35, we finished observing Volta. The chimpanzee party had already moved several hundred meters away and Volta was left alone. At the last location, Volta leaned approximately 50 m away from his crash site.

The day after the accident, September 3, 2022, at 12:35, we began following this party. However, Volta and Nkombo were not spotted within this party. We conducted field research of the M group until September 5. On all three days, we encountered parties of approximately the same size as on the day of the accident, and although almost all adult males were found in the party, Volta and Nkombo were not spotted within the parties.

M.S. left Mahale on September 6. The local assistants did not encounter Volta in the group for several weeks. On October 17, 2022 (45 days after the accident), an assistant reported that Volta was found in a party with Nkombo and that Volta appeared healthy. Assistants have since confirmed that Volta has been healthy until at least July 2023.

### Social context and behavior of chimpanzees to Volta

**Figure 6:**
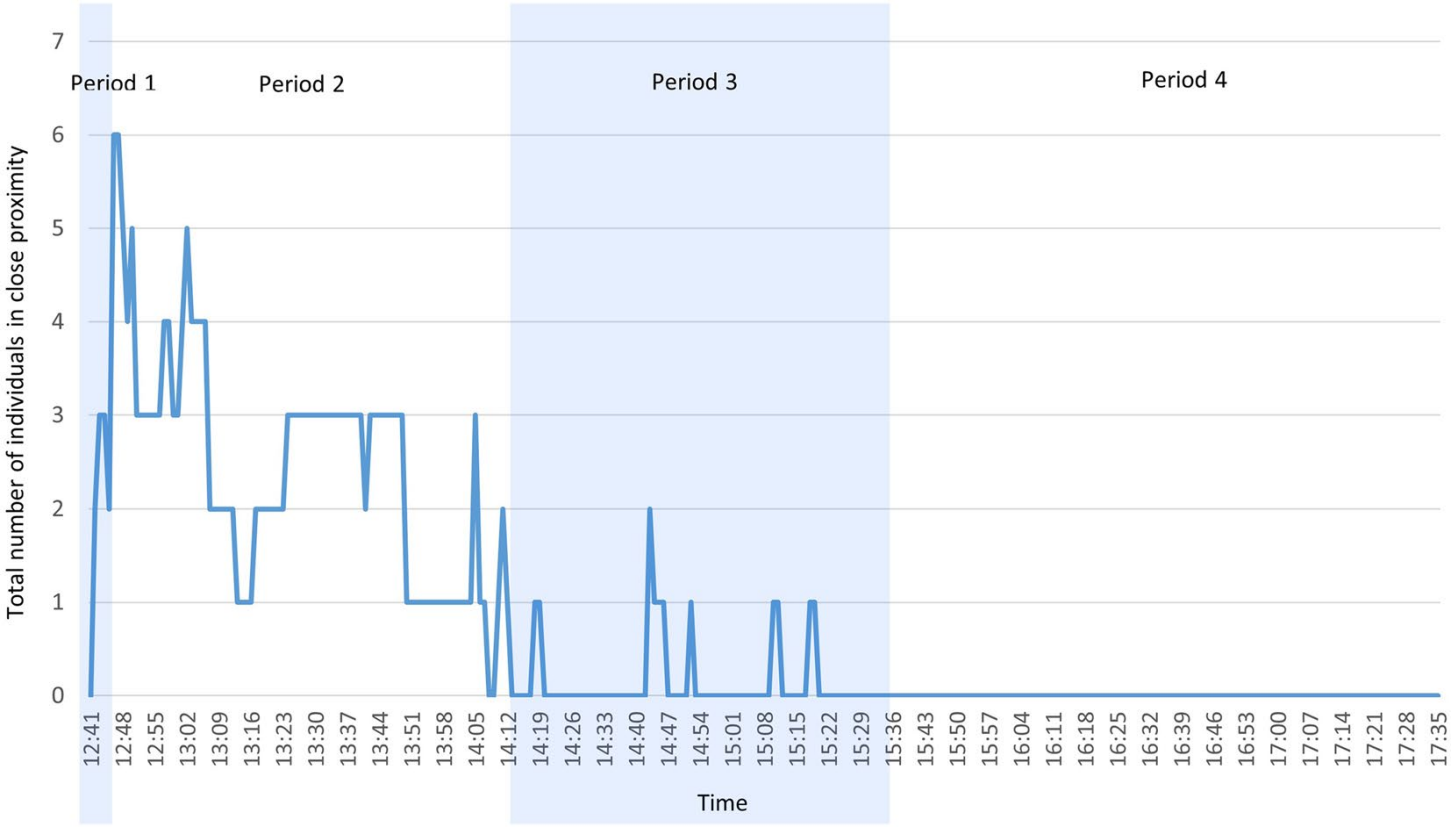
Changes in the number of individuals in close proximity to Volta from the time of the fall accident (12:41 p.m.) to the end of the observation (17:35 p.m.) in 1-min intervals. The blue and white backgrounds represent changes in social context, with Period 1 up to Teddy’s arrival, Period 2 up to Teddy’s departure, Period 3 up to Orion’s departure, and Period 4 up to the end of observation.

The social context around Volta after the accident was divided into four periods (Fig. 6). Period 1: from immediately after the accident to the time when the members, including the alpha male Teddy, arrived (12:46); Period 2: from the time when Teddy arrived to the time when most of the members including Teddy left (14:11); Period 3: from the time when Teddy left to the time when Orion, who remained near Volta, left (15:34); and Period 4: from the time when Orion left and Volta was left alone to the end of the observation period (17:35). The mean (± SD) number of individuals in close proximity during Periods 1, 2, 3, and 4 were 2.0 ± 1.1, 2.5 ± 1.3, 0.2 ± 0.4, and 0.0 ± 0.0, respectively. Periods 1 and 2 had more individuals in close proximity to Volta than Periods 3 and 4 (Kruskal–Wallis test: χ^2^ = 242.62, df = 3, p < 0.001, η^2^ = 0.825). The maximum number (six animals) was reached immediately after the arrival of Teddy.

Of the 26 individuals, except for Volta, observed on the day of the accident, 14 were in close proximity to Volta at least once between the occurrence of the accident and end of the observation period (Table 1). According to sex and age, the number of individuals that stayed in close proximity to Volta and their mean (± SD) duration of proximity were 8 of 9 adult males (21.9 ± 26.5 min), 1 of 3 young males (0.33 ± 0.47 min), 2 of 3 juvenile males (0.67 ± 0.47 min), 3 of 8 adult females (4.5 ± 10.8 min), and 0 of 1 juvenile female (except for 3 infants that were constantly with their mothers). When comparing adults alone, more males were in close proximity than females (Fisher’s exact test: p = 0.0498). Significant differences in the duration of proximity were found among the sex and age categories, with adult males staying in close proximity to Volta for longer periods of time than individuals in the other categories (Kruskal–Wallis test: χ^2^ = 10.337, df = 4, p = 0.035, η^2^ = 0.449; Table 2).

**Table 1:** Twenty-seven individuals observed on the day of the accidental fall of Volta and the number of units each individual was in close proximity to Volta for (min).

Name	Age	Sex	Number of units being in close proximity to Volta
Carter	Adult	Male	4
Darwin	Adult	Male	10
Primus	Adult	Male	17
Orion	Adult	Male	76
Christmas	Adult	Male	18
Emory	Adult	Male	2
Teddy	Adult	Male	64
Ichiro	Adult	Male	6
Azam	Adult	Male	0
Figaro	Young	Male	0
Omaly	Young	Male	1
Zamma	Young	Male	0
Volta	Juvenile	Male	NA
Peace	Juvenile	Male	0
Ryoma	Juvenile	Male	1
Oz	Juvenile	Male	1
DV22	Infant	Male	0
Devota	Adult	Female	0
Effie	Adult	Female	2
Koopy	Adult	Female	0
Nkombo	Adult	Female	1
Omo	Adult	Female	0
Puffy	Adult	Female	0
Teto	Adult	Female	0
Christina	Adult	Female	33
Daisy	Juvenile	Female	0
KP20	Infant	Female	0

**Table 2:** List of behaviors identified during the proximity to Volta. Numbers are the total unit minutes during which individuals in close proximity to Volta for more than 10 min were confirmed to have performed each behavior.

Behavior	Commonality	Orion	Teddy	Christina	Christmas	Primus	Darwin	Total
Stay nearby/visitation	Common	18	34	20	5	8	6	91
Manipulate/touch	Common	3	3	7	3			16
Rough treatment	Common		2					2
Peer	Common	7	5	3	6	2	2	25
Display	Common		6				2	8
Touch	Common	3	3	7	3			16
Sniff	Common	5	8		4	2	1	20
Groom	Occasional	42	15		3	7		67
Warning call	Occasional							
Distress call	Occasional							
Attack	Rare							
Prevents access	Rare							
Stunned silence	Rare							
Swats flies	Rare		5		1		2	8
Cannibalism	Rare							
No reaction	Rare							
Mobilize	Rare							
Lick	None	7	4	2	3			16
Search for objects with blood	None	4	2	2	5			13
Bite softly	None		6					6

Five adult males and one adult female were in close proximity to Volta for more than 10 min (Table 2). The common behaviors found in these six individuals during proximity to Volta were staying nearby/visitation and peeking. Sniffing, licking, and searching for objects with blood were clearly concentrated at the beginning of Periods 1 and 2, whereas grooming and staying nearby/visitation dominated the rest of the periods. Aggressive behaviors such as rough treatment and display behavior were observed only in Teddy and Darwin, whereas affiliative behaviors such as licking and biting softly were observed only in Teddy. Social grooming is the other typical affiliative behavior, and multiple adult males Teddy, Orion, Christmas, and Primus groomed Volta (Table 2).

### Symptoms of Volta and cause of fall accident

We confirmed that Volta avoided charging display by Ichiro on the ground at 12:37 p.m. by running up a tall tree of *P. microcarpa*, and fell shortly thereafter.

When Volta occasionally opened his mouth, we observed dentition and no dental damage. In addition, although blood was on Volta's face at the beginning of the observation of the accident (Fig. 1), bleeding was only observed from both nostrils. No trauma, including abrasion, was observed throughout the entire body of Volta. We observed bloody vomiting from the oral cavity at 13:38 and 14:10. Subsequently, at 16:02, 16:14, and 17:28, Volta vomited stomach contents that did not contain blood (Fig. 5). This suggests that the vomiting was not due to an internal stomach ulcer and that the bloody vomiting was either bleeding from the interior of the nasal cavity or the blood that had temporarily accumulated in the oral cavity.

Immediately after the accident, the spilled blood pooled near Volta’s eye sockets, but he was blinking. While Volta was lying on his side, both hands grasped nearby vines. When Carter approached at 12:45, Volta grabbed his hair near the face with left hand and pulled him. Volta showed grimaces and emitted pant-grunts toward adult males. At 12:48, Volta walked quadrupedally and continued to act voluntarily, albeit unsteadily and intermittently. This suggests that Volta's consciousness was restored at least a few minutes after the accident, although Volta’s loss of consciousness immediately after the accident can be determined only by external observation.

Volta was not observed for some time after the day of the accident but continued to travel with Nkombo after he was reconfirmed in the group, and no physical impairment was observed. This suggests that all the symptoms of Volta caused by the accident were temporary.

Combining all the observational information, we inferred the following: Volta accidentally fell while running up a branch of a tall tree of *P. microcarpa* to avoid charging display by Ichiro. There were no shrubs, vines, or other vegetation between the branch and the ground that could soften the impact of the crash, and thus, we assume that Volta landed directly on the ground. He may have landed quadrupedally, but the acceleration of his landing caused his face to strike the surface of a log, fracturing the nasal bone in the nasal cavity and simultaneously causing a strong concussion. From immediately after the accident to the end of the observation period, we observed convulsions or fidgeting every few seconds for several tens of seconds. In addition, throughout the observation period, Volta walked unsteadily, and could not maintain a sitting posture on the ground easily. These symptoms are consistent with the temporary symptoms caused by a strong concussion.

## Discussion

On the day of the accident, Volta was a member of a party that included all adult males and travelled with his adoptive mother, Nkombo. Fourteen of the 26 party members, except for Volta, recorded on this day approached Volta in close proximity after the accident. Adult males were more frequently and longer in close proximity to the Volta. For example, Darwin and other adult males, who had been traveling with the alpha male, Teddy, were at a distance from the crash site during Period 1, but immediately approached and maintained close proximity to Volta as soon as they discovered that Volta was lying down. In addition, another adult male, Azam, never approached, and Nkombo approached within 1 m of Volta for only one unit (1 min), but they all maintained a distance of 3–5 m between Periods 1 and 2 while watching Volta from the tree. These observations suggest that the anomalies that occurred among the party members were of strong interest to almost all other members, who each tried to confirm the status of Volta by performing animacy detection^10,18^ through exploratory behavior toward Volta.

This also suggests that the social relationships among chimpanzees around Volta are inseparable, as individuals of multiple sex–age categories were densely concentrated in areas close to Volta. Chimpanzees form fission–fusion societies in which adult males have stronger social influence than females or immature individuals^19,20^. Social rank of adult males is higher than that of other sex–age categories and an alpha male has an extremely strong status among adult males^21^. When multiple individuals congregate in a small area, social tension is likely to increase, promoting charging display and aggressive behavior in males^22^. In this case, we observed multiple charging displays exhibited by Teddy, Darwin, and other adult males around Volta. For example, an adult male, Carter, who was in close proximity to Volta during Period 1, left Volta when Teddy approached. In addition, after the accident, Nkombo watched Volta from a distance of 3–5 m, but was not within 1 m. However, they resumed traveling with Volta after his recovery. These observations suggest that the social situation surrounding Volta created by alpha and other males prevented Carter and Nkombo from being close to Volta, although neither exhibited stress-related behaviors such as showing grimaces or self-scratching, even when not in close proximity to Volta.

We observed affiliative behaviors, such as staying nearby, peering, manipulating/touching, sniffing, and grooming, as well as aggressive behaviors, such as rough treatment and display (around Volta), in individuals in close proximity to Volta (Table 2). Individual variations were large. For example, Teddy engaged in prolonged grooming, and Orion repeatedly waited for Volta, who was unable to walk normally after most party members had moved away with Teddy, in addition to affiliative behaviors. Behaviors reported as “commonly” observed in previous studies on the behaviors of individuals in close proximity directed towards collapsed and inanimate subjects, such as stay nearby/visitation, rough treatment, peer, display, touch/manipulate, and sniff^10^ were observed in all six individuals in this case. Among the behaviors reported as “occasionally” and “rarely” found, we observed grooming and swatting flies. These results suggest that the behaviors toward Volta exhibited by individuals in his close proximity were, overall, similar to those observed towards collapsed and inanimate subjects.

However, licking (Volta’s blood) and biting softly, which have not been reported as behaviors toward collapsed and inanimate subjects, were observed toward Volta. Chimpanzees show curiosity about new injuries in other individuals and perform inspecting behavior, as well as grooming and licking wounds^2,12,23^. Volta was temporarily collapsed by definition when discovered by M. S., but even then multiple adult males licked Volta's blood. However, although in an abnormal state of loss of animacy, Volta raised his head only a few minutes after M. S. noticed him lying on the ground, convulsing, and in this sense, it was obvious for the other chimpanzees that Volta was animate. In addition, Volta and other members of the party were traveling together right up to the moment of the accidental fall. They also had the opportunity to be in close proximity to Volta after the accident and observe his recovery process. In these circumstances, this case that many chimpanzees licked Volta’s blood from his body and softly bit Volta’s fingers is considered to be consistent with other reported cases of chimpanzees’ reactions to injured but apparently animate individuals^2,12,23^.

Young male chimpanzees may be subjected to ostracism, such as being attacked or ostracized by other group members, if they do not emit pant-grunts toward the alpha or other adult males^24^. It is also important for immature individuals to make other adult males recognize their presence and judge their tolerance by emitting pant-grunts^25^. The facts that Teddy groomed Volta for extended periods, that Orion waited a long time for Volta to travel, and that several individuals licked Volta’s blood indicated tolerance and consideration in adult males for a juvenile male that was still in an abnormal state and that Volta did not refuse, but was unable to emit pant-grunts at them, even though they recognized that he was recovering. Since Volta's accident occurred immediately after the two-year absence of researchers due to the coronavirus, quantitative data on the relationship between Volta and other individuals before the accident occurred is not available. However, during this period, assistants have confirmed that Volta lost its mother Vera, and Nkombo subsequently became his adoptive mother, and that there is no evidence that any particular adult male had a close relationship with Volta. The kinships between all adult males and juveniles in the M group, including Volta, are unknown, and it is rare for adult males to actively groom such juveniles or wait for them to travel^2,26^. Thus, the intensive affiliative behaviors toward Volta by adult males are considered to be temporary behaviors caused by Volta’s abnormal state due to the fall accident.

Recently, there has been much debate on how chimpanzees perceive death^9,10^. This study provides rare but valuable wild data that contribute to this debate. For wild chimpanzees, the health status of others, whether alive or dead, or soon to die or recover, should be recognized and understood in a continuous gradient of temporal change, and not in a binary discontinuity of alive or dead^17,18^. The analysis of this study suggests that the chimpanzees’ responses to a recently dead body that have been reported to date are an extension of their responses to collapsed and inanimate subjects or subjects in recovery processes, and in this sense, do not provide evidence that they are “aware of death.” In Period 1, Volta was lying on his back and convulsing, in Period 2, Volta sat immediately after Teddy approached, and in Period 3, Volta began to walk. Orion was the only individual who has been in close proximity to Volta through Periods 1–3, and Orion groomed Volta in Period 2 and 3, but not in Period 1, and in Period 3, Orion repeatedly shook a vine to wait for Volta. Chimpanzees may groom not only injured but clearly animate individuals^2,12^, but also clearly inanimate or dead individuals^1,9^. It is suggested that the temporal change in Orion's affiliative behavior to Volta was not determined by the dichotomy of whether the victim was alive or dead, but occurred according to the changes in the state of Volta who has lost his animacy. Accumulating observations of not only how individuals respond to dead bodies, but also of the behavior of surrounding individuals toward individuals who are alive but suffering from loss of animacy and are unable to move normally are important to the discussion of the perception of death in chimpanzees and other non-human animals.

## Materials and methods

We conducted a 6-day study on the behavior of chimpanzees of the M group in the Mahale Mountains National Park, Tanzania, from August 31 to September 5, 2022, during the dry season, for a total of 32.3 h^12,27^. The exact size of the M group was unknown at the beginning of the research because it was conducted immediately after the absence of researchers owing to the COVID-19 pandemic, but the M group was estimated to have consisted of approximately 60 members (Dr. M. Nakamura, pers. comm.). The study period coincided with a period when chimpanzees relied on the liquid fruit of *P. microcarpa*, a major food species for chimpanzees of the M group^27^, to form relatively large parties, and the mean party size (± SD) during this period was 26.2 ± 2.1 individuals^19^. Age categories were defined as follows: the infant was defined 0–4 years and the juvenile was defined as 0–8 years of age for both sexes. Young males were defined as 9–15 years old and young females as 9–12 years old. Adult males were 16 years of age or older, and adult females were 13 years of age or older^20^.

On September 2, 2022, the day of the fall accident, we observed 27 individuals (nine adult males, eight adult females, three young males, four juvenile males, one juvenile female, one infant male, and one infant female), and all the adult males of the M group were gathered. At 9:20 a.m. on September 2, after finding a member of the M group, we began to follow an old female, Nkombo, as a target individual to observe her behavior. At the start of the observation, Nkombo traveled with an unrelated 5-year-old juvenile male, Volta. During the approximately two years that the researchers were away, Volta’s mother, Vera (VR), disappeared and was presumed dead. An assistant who continued to observe Volta during the COVID-19 pandemic confirmed that orphaned Volta always traveled with Nkombo (Mr. Abdalah R., pers. comm.). The fact that Nkombo always traveled with Volta is thought to be an adoptive behavior by Nkombo^28,29^. At the beginning of the observation, Volta presented no health or mobility problems.

The fall accident of Volta was estimated to have occurred at 12:37 p.m. Up to that point, we had been following and observing Nkombo. However, after becoming aware of the accident, we switched observations to thoroughly record Volta’s condition and the events that occurred around Volta. We primarily used the video function of a digital camera (SONY DSC-RX10M4), photography, and field notes to record observations. After we finished the field research on September 5 and returned to Japan, we contacted assistants and asked them to gather information on the whereabouts of the Volta after the accident.

We defined “close proximity” as being within 1 m of Volta’s body, and used a one-zero sampling method with one minute as one unit to examine the number of individuals who were in close proximity to Volta for each unit and the number of units (minutes) that each individual was in close proximity to Volta for. The length of time (number of units) in close proximity to Volta for each age category and the number of individuals in close proximity to Volta for each social context was compared using the Kruskal–Wallis test. The free statistical software HAD (version 17) was used for analysis^30^. Individuals who were in close proximity to Volta for more than 10 min were considered long-time attendants. We examined their behaviors toward Volta during their close proximity to Volta and counted the number of units (minutes) during which each attendant directed each behavior toward Volta.

To obtain findings on Volta’s condition from a medical perspective, we visited the Department of Biology, National Defense Medical College, accompanied by video data, photographs, and behavioral observation records. While checking the videos and photographs with others, we diagnosed Volta’s symptoms.

### Ethical note

To conduct field research on wild chimpanzees in Mahale, we complied with the protocols approved by the Tanzania Wildlife Research Institute (TAWIRI) and adhered to the legal requirements of the government of Tanzania. Our research adhered to the American Society of Primatologists (ASP) Principles for the Ethical Treatment of Non-Human Primates and was approved by the Ethics Committee for Animals of Teikyo University of Science (Approval No. 22C024).

## Data Availability

The video data supporting the findings of this study are openly available in Science Data Bank, at https://www.scidb.cn/en/s/iMvyEr, https://doi.org/10.57760/sciencedb.09546.
